# Editorial: Neurosociology: A New Field for Transdisciplinary Social Analysis

**DOI:** 10.3389/fsoc.2022.911361

**Published:** 2022-04-26

**Authors:** Vincenzo Auriemma, Gennaro Iorio, Rosalba Morese, Rudina Rama

**Affiliations:** ^1^Department of Political and Social Study, University of Salerno, Fisciano, Italy; ^2^Faculty of Communication, Culture and Society, Università della Svizzera italiana, Lugano, Switzerland; ^3^Faculty of Biomedical Sciences, Università della Svizzera italiana, Lugano, Switzerland; ^4^Department of Sociology, University of Tirana, Tirana, Albania

**Keywords:** sociology, neurosociology, transdisciplinary, empathy, social psychology

## Introduction

Within this research theme, it was possible to open a new transdisciplinary Frontier, within which sociology and neuroscience in general, can contribute to the improvement of social researchers and all interested scholars, expanding the different levels of analysis of the social sciences. The key concept, from the classics of sociological thought, is based on a discipline that can contain this cultural transformation initiated by the discovery of mirror neurons forward, namely neurosociology. The latter must be considered as the discipline that investigates social interactions and socialization with respect to the structures and functions of the brain. As a transformative perspective that builds on the postulates of sociology and, therefore, whose goal is not to favor one discipline over another, it falls within the trappings of a discipline that aims to investigate how the human brain influences the complicated set of forces that drive human relationships and social organization. However, the distinctiveness of this approach also warrants the analysis of neurosociology within social processes and how they influence neural function. Therefore, neurosociology is closely related to sociology underlying the socio-cultural-interactionist derivation of every behavior. The contributions collected, therefore, range from bibliographic and purely theoretical reviews, up to empirical research, the set of products, collected within this call, will allow expanding the field and, above all, open to trans-disciplinarity.

## Review and Opinion Contributions

The purpose of the review by Saladino et al. was to highlight, through empirical data contained within the bibliography, from the past 5 years, focusing interest on the connection between empathy and neuroscience. In this regard, the authors presented an in-depth study related to the social context involving violent criminals and psychopaths. The intent was to assume that empathy is an important to feel and understand the cognitive and emotional states of self and other people. Reflecting on this issue allowed empathy to be considered a psychological concept as well as a sociological one.

Reading Firat's text, it is possible to note that negative experiences, such as discrimination and exclusion, can influence health increasing physiological responses to stress, both physical and mental. Adding to the rich contribution provided to us is the ability to fit within the literature that until now has been lacking a sociological vision that could account for biological processes that are structurally and culturally shaped.

The study proposed by Shkurko's is characterized by the analysis of the natural effectiveness of the face-to-face medium. This idea, based on face-to-face communication, brings to the reader's attention the evolutionary mismatch hypothesis that evolved human nature is dependable on all other kinds of device-mediated communication that might cause negative interactions.

To better understand the relationship between social experiences and the brain, it is important to investigate neuroscience together with the social sciences. The other work, which arouses a lot of interest, is that proposed by Racionero-Plaza et al. who highlighted how the environment, and more specifically social experience, can influence the brain and even its genes. However, to fully understand these mechanisms, trying to understand what kinds of social interactions lead to certain outcomes in the brain, which differ from person to person and in behavior, neuroscience requires the postulates of the social sciences. This aspect allows for those inputs needed by neuroscience to enrich the meaning toward new discoveries about the brain.

A completely new aspect in this context, is the work proposed by Borbón, in fact through his considerations it is possible to begin to explore a topic of very close relevance, namely criminal abolitionism, which, in the neuroscientific context, takes the name of criminal neuroabolitionism. This new approach is based on the discoveries made in various disciplines, especially in the field of neuroscience, to provide a new perspective. In this sense, the transdisciplinary aspect that emerges is strong, and the link between sociology and social neuroscience is clear, offering several starting points for future studies: (1) on the neuropsychological effects of incarceration; (2) the contribution of neuroscience to investigate criminogenic social factors; (3) a new point of view to better understand criminal law as a mechanism of social control.

The trans-disciplinary openness of our call prompted Sipes et al. to provide us with interesting insights into the topic of adolescence, thus an issue that is increasingly embedded in everyday life, both within legislation and as an object of inquiry. The authors, in this paper, highlight the phases of neural and social development, basing themselves on and analyzing prosocial decisions, ascertaining that these are beneficial for personal and social wellbeing. A review highlighting the progress made in this field was necessary, reporting a total of 25 articles. In this regard, the authors offer predictions for testing a new model, proposing new future directions for studying prosocial behavior.

Finally, among the opinion articles, there is an interesting view regarding the COVID-19 emergency that seems to have passed its acute phase. Within the Paradisi et al. text, one can read the emergence of the role of online digital technologies. The authors describe, in a timely manner, how the increase in online social interactivity has increased its activity due to the social distancing to which we have been forced. However, it has had negative effects due to emotional and even physical isolation, as what has been missing is body language, which has always played a crucial role in face-to-face social relationships. This epochal change has affected the wellbeing of people, assuming very serious effects in the long run, affecting, above all, the population affected by specific fragilities. The goal of the authors was to encourage new constructive reflections, with the aim of increasing awareness of these topics of close relevance.

## Empirical Results: Quantitative Data and Original Research

The section on empirical works opens with a work offered to us by Alfonso and Molano, the authors accurately present the work done by the musician John Cage, who based his art on “sound-scene-vision” experimentation within the theater, media, and art. The study is presented with different readings, one certainly regards the “innate rules of perception” and the other brings out the interesting relationship between art and neuroscience.

The aim of the study by Yang et al.'s, was to explore how the emotional expression of news in the post-truth era affects the cognitive processing of the audience. Indeed, news written with several kinds of emotional expression rather than neutral, can lead to changes in the cortical activity of the people belonging to the investigated sample, who were examined with electroencephalograms, and basing it on the method of functional brain connectivity. The results showed that emotional speech involved more cortical brain activity.

The article by Poali et al., describes qualitative research based on tears of joy, an emotional element of great prominence, and that, in the sociological and anthropological literature, is very present, for example in the ritual weeping of Ernesto de Martino. This research, consisting of semi-structured interviews, was carried out in India and Japan, processing the qualitative data extrapolated from the transcripts, through the software MAXQDA, to investigate this dimorphic emotional expression. The work is based on the hypothesis that tears of joy are not only an atypical expression due to what the authors call “super joy,” but rather an emotional experience with a specific adaptive function.

Finally, concluding our review is the work of Zheltyakova et al. They present work investigating the characteristics of provocative behavior in an environment where the subject is anonymous. However, the personal behavior of the “anonymous” person during the processing of the provocative feedback remains largely unknown. The fMRI study conducted, has led to the evidence that through Taylor's aggression paradigm and, within a game that can generate “social provocation,” the neural system activates, as a compensatory mechanism during social interactions, a system capable of dropping socially relevant information.

## Conclusion

The main goal of this call, which we felt was fully met, was to highlight some of the insights that can be gleaned from a transdisciplinary exploration that can both account for the postulates of sociological thinking and adapt them to new neuroscientific paradigms. This has allowed, in this way, to avoid a drift into the two biggest reductionisms, cultural and biological, to put in evidence not to lean toward one discipline rather than another, but that all disciplines, in a trans-disciplinary agreement, can generate a greater knowledge for each of them (e.g., Auriemma et al., 2020; Fante et al., 2022). Consequently, one should be wary of any theory that claims that our understanding of others is only a matter of biological input, as the cultural interaction aspect remains at the core of any discourse. The sciences need as much transdisciplinarity as possible in this field, maintaining personal objectives that cover the variety of skills and strategies we draw on, but, above all, contributing to a common knowledge that can place the person at the center of any scientific discourse, avoiding scientistic reductionism and allowing researchers and scholars to draw from the broadest possible sources and that we use to understand and make sense of others. To conclude, the wonderful and ambitious goal that we editors, belonging to distant disciplines, had set ourselves and that masterfully the authors of each paper have been able to satisfy, we must emphasize that today it is possible to go beyond what is directly available if we want to reach deeper levels of interpersonal understanding. Given the polysemous nature of the various notions presented in this appeal, one might naturally wonder if it would not be better to explore transdisciplinarity more thoroughly throughout the research articles.

## Author Contributions

All authors listed have made a substantial, direct, and intellectual contribution to the work and approved it for publication.

## Conflict of Interest

The authors declare that the research was conducted in the absence of any commercial or financial relationships that could be construed as a potential conflict of interest.

## Publisher's Note

All claims expressed in this article are solely those of the authors and do not necessarily represent those of their affiliated organizations, or those of the publisher, the editors and the reviewers. Any product that may be evaluated in this article, or claim that may be made by its manufacturer, is not guaranteed or endorsed by the publisher.

## References

[B1] Auriemma V. Iorio G. Roberti G. Morese R. (2020). Cyberbullying and empathy in the age of hyperconnection: an interdisciplinary approach. Front. Sociol. 5, 551881. 10.3389/fsoc.2020.551881 33869494 PMC8022687

[B2] Fante C. Palermo S. Auriemma V. Morese R. (2022). Social Inclusion and Exclusion: How Evolution Changes Our Relational and Social Brain. London: IntechOpen.

